# Monkey in the middle: why non-human primates are needed to bridge the gap in resting-state investigations

**DOI:** 10.3389/fnana.2012.00029

**Published:** 2012-07-26

**Authors:** R. Matthew Hutchison, Stefan Everling

**Affiliations:** ^1^Graduate Program in Neuroscience, Western UniversityLondon, ON, Canada; ^2^Department of Physiology and Pharmacology, Western UniversityLondon, ON, Canada; ^3^Robarts Research Institute, Western UniversityLondon, ON, Canada; ^4^Brain and Mind Institute, Western UniversityLondon, ON, Canada

**Keywords:** resting-state, non-human primate, functional connectivity, macaque, animal model, spontaneous activity, functional MRI (fMRI)

## Abstract

Resting-state investigations based on the evaluation of intrinsic low-frequency fluctuations of the BOLD fMRI signal have been extensively utilized to map the structure and dynamics of large-scale functional network organization in humans. In addition to increasing our knowledge of normal brain connectivity, disruptions of the spontaneous hemodynamic fluctuations have been suggested as possible diagnostic indicators of neurological and psychiatric disease states. Though the non-invasive technique has been received with much acclamation, open questions remain regarding the origin, organization, phylogenesis, as well as the basis of disease-related alterations underlying the signal patterns. Experimental work utilizing animal models, including the use of neurophysiological recordings and pharmacological manipulations, therefore, represents a critical component in the understanding and successful application of resting-state analysis, as it affords a range of experimental manipulations not possible in human subjects. In this article, we review recent rodent and non-human primate studies and based on the examination of the homologous brain architecture propose the latter to be the best-suited model for exploring these unresolved resting-state concerns. Ongoing work examining the correspondence of functional and structural connectivity, state-dependency and the neuronal correlates of the hemodynamic oscillations are discussed. We then consider the potential experiments that will allow insight into different brain states and disease-related network disruptions that can extend the clinical applications of resting-state fMRI (RS-fMRI).

## Introduction

The human brain is a system composed of multiple levels organized into integrative network configurations. At a gross topological scale, spatially distributed, interconnected brain areas interact to perform circumscribed functions—communicating via patterns of synchronization presumably supported by long-range white matter fiber tracts (for reviews see Buzsáki and Draguhn, 2004; Buzsáki, 2006; Bullmore and Sporns, 2009; Bressler and Menon, 2010; Sporns, 2010; Breakspear and McIntosh, 2011). Through the evaluation of coherence in spontaneous low-frequency fluctuations (0.01–0.1 Hz) of the blood-oxygenation-level-dependent (BOLD) signal, resting-state fMRI (RS-fMRI) has proven to be a valuable tool for characterizing these functional relationships. While the term “state” implies a well-controlled period of investigation, the “resting-state” is simply a period of recording in the absence of any explicit task paradigm. Investigations are not time-locked to specific task-stimuli and subjects are free to cycle through normal cognitive processes while passively lying awake in the scanner with eyes open and fixating or closed. Owing to the non-invasive nature of the technique and its ease of implementation, the vast majority of an ever-growing field of studies examining intrinsic brain activity and resting-state functional connectivity has been primarily conducted on human subjects. Great strides have been made in furthering our understanding of large-scale brain topology, including the identification of multiple, anatomically distributed resting-state networks (RSNs) (Biswal et al., 1995; Beckmann et al., 2005; Damoiseaux et al., 2006) that are involved in processes that cover a broad range of lower- and higher- order processes. Further motivation for the use of RS-fMRI has come from the discovery of RSN alterations across a spectrum of disease states (Auer, 2008; Greicius, 2008; van den Heuvel and Hulshoff Pol, 2010).

Given the notable successes of this relatively new area of neuroimaging (Biswal et al., 1995), the field is rapidly advancing toward more complex characterizations of brain architecture and potential clinical applications. The goal of this review, however, is to caution against premature conclusions being drawn when there are a number of crucial open questions concerning both the technique and interpretation of RS-fMRI. For example, the evolution of network topology, the neural origins underlying spontaneous BOLD activity, the potential function of such activity, the effectors of disease-related changes of the signal correlations, and the correspondence between functional and structural connectivity all remain unresolved albeit essential elements in making inferences from resting-state findings. While there are human studies attempting to examine each of these issues, they are limited by ethical or practical restrictions that preclude the invasive experimentation necessary to directly assess these questions. Therefore, we contend that the examination and manipulation of the functional brain organization in animals is a requisite for forthcoming resting-state research as they allow these necessary experimental manipulations to be carried out. While rodent models will serve an integral role in these studies, we advocate for the use of non-human primates as the most suitable model of human brain networks, discussing ongoing work establishing homologies between the species, outlining relevant contributions of macaque resting-state investigations to elucidating the aforementioned open questions, and discussing critical future experiments.

## Comparative approach to neurobiology

Beyond the intrinsic motivation to explore and classify species, comparative biology can significantly enhance our understanding of mammalian brain organization and evolution through cross-species evaluation of homologies. Determining the relationships among cortical areas and networks between species can be difficult because they differ in both brain and body size, the relationship of which is nonlinear (Van Dongen, 1998). Over the course of evolution, brain regions can duplicate, fuse, reorganize, or expand, changing the proportions of different regions as well as its microstructure and connectivity (Sereno and Tootell, 2005; Hill et al., 2010). The task of comparison is made more difficult because the measurement techniques are often different between species. RS-fMRI circumvents this final limitation and provides a more direct tool for cross-species comparisons as the same methodology can be used to directly compare species because there is no task requirement. By determining which features are conserved across species it can indicate brain regions and patterns that have a basic functional and/or developmental role. The results can also help validate previously extrapolated findings derived from animal models (e.g., disease or knockout models) that afford a greater range of experimental manipulations not practical or possible in humans. However, in comparison to the exponential growth of resting-state publications in humans (Snyder and Raichle, 2012), RS-fMRI investigations of other species is considerably lacking. To date, published reports have included the mouse (Jonckers et al., 2011), rat (Lu et al., 2007; Pawela et al., 2008; Hutchison et al., 2010; Liang et al., 2011), macaque (Vincent et al., 2007; Margulies et al., 2009; Hutchison et al., 2011), and chimpanzee (Rilling et al., 2007). While these studies have begun to expand our knowledge of unique and preserved network properties, a greater range of animals within and between evolutionary branches needs to be examined, capitalizing on the strength of RS-fMRI.

### Selecting an animal model

Of the limited number of species examined with RS-fMRI, the rat and macaque, an old-world monkey, represent the largest proportion of animal investigations, partially owing to their widespread and ongoing contributions across multiple areas of neuroscience. It is well established that macaque and human brains share a high degree of similarity in terms of cytoarchitecture (Petrides and Pandya, 1999, 2002; Ongür et al., 2003), functional organization (Rees et al., 2000; Koyama et al., 2004; Petrides et al., 2005; Nakahara et al., 2007), anatomical connections (Croxson et al., 2005; Kelly et al., 2010) and as will be discussed, RSN topology. From a practical perspective, however, their usage is made difficult by constraints such as their cost, handling procedures, housing requirements, and gestation time. Rodents circumvent some of these limitations and at first glance, appear to be a well-suited animal model for comparisons of network topology.

A rodent brain shows less inter-subject variability than that of monkeys in terms of morphological, anatomical, and functional attributes. Consequently, it is easier to induce reliable lesions and interpret findings in the smaller rodent brain. Further, the majority of behavioral and pharmacological experiments are performed in rats, providing a strong theoretical foundation and transgenic mice have provided multiple models to study normal and abnormal brain systems. The analysis of the networks of the rat using RS-fMRI has consistently revealed multiple cortical and subcortical networks composed of contralateral homologs in both awake and anesthetized animals (Pawela et al., 2008; Hutchison et al., 2010; Zhang et al., 2010b; Liang et al., 2011; Jonckers et al., 2011). Bilateral homologous connectivity has also been observed to a lesser extent in mice (Jonckers et al., 2011). How do these configurations correspond to primates?

**Figure 1:**
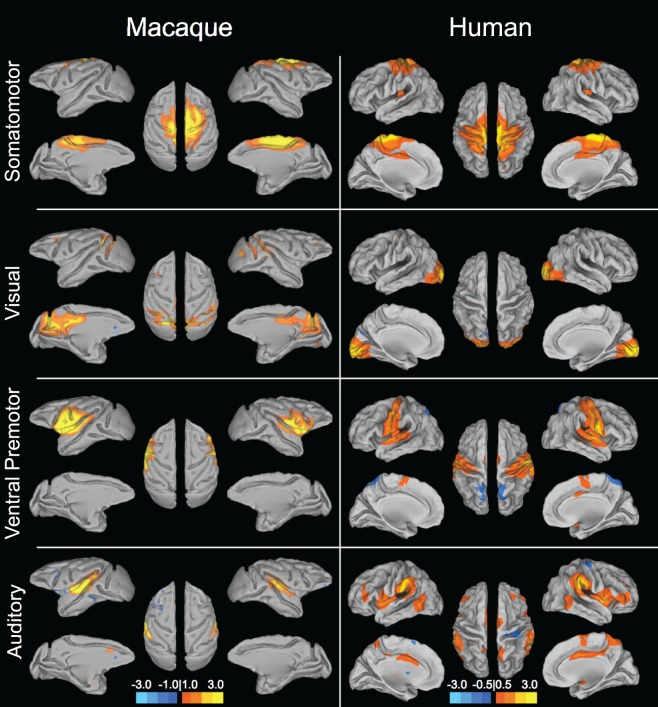
**Sensory and motor resting-state networks of the macaque (left column) and human (right column) showing connectivity between bilateral homologs.** Putative functional roles of the networks are indicated on the left. Macaque networks reproduced with permission from Hutchison et al. (2011). Human connectivity maps (*N* = 12) derived from ICA of data from Hutchison et al. (2012).

**Figure 2:**
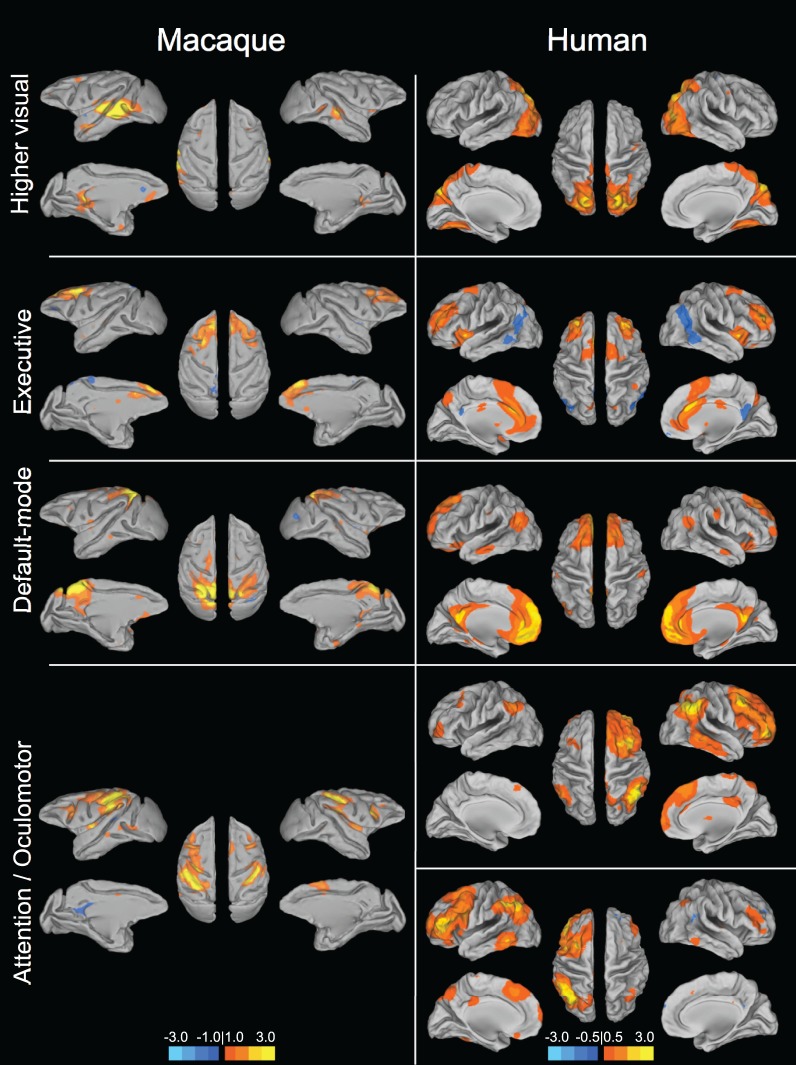
**Homologous higher-order resting-state networks of the macaque (left column) and human (right column).** Putative functional roles of the networks are indicated on the left. Macaque networks reproduced with permission from Hutchison et al. (2011). Human connectivity maps (*N* = 12) derived from ICA of data from Hutchison et al. (2012).

Human and non-human primates do show strong functional connectivity between interhemispheric homologs in what may be regarded as lower-order sensory and motor networks (Figure 1). Higher-order networks are also most often composed of hierarchically organized symmetric pairs of regions (Abou-Elseoud et al., 2010; Yeo et al., 2011; Figure 2). Homologous synchronization is even present in newborn infants (Fransson et al., 2007; Gao et al., 2009). The results suggest that interhemispheric synchronization of low frequency BOLD fluctuations is phylogenetically preserved across mammalian species and may underlie a rudimentary aspect of brain function that can be sufficiently captured in a rodent model. There have already been successful applications of rat RS-fMRI, for example directly studying cortical reorganization (Pawela et al., 2010; van Meer et al., 2010), exsanguination (Kannurpatti et al., 2008), dose-dependent anesthetic effects (Lu et al., 2007; Wang et al., 2011), and an extensive range of pharmacological manipulations (Bifone et al., 2010). Therefore, rodent models can serve a prominent role in advancing the resting-state field by providing a robust, economical, and manipulatable model of brain architecture and dynamics.

However, limitations of rodent models emerge when one attempts to study the relations among more distributed areas or higher-order cognitive abilities that shift the selection of the optimal model to non-human primates. There are over 80 million years separating rodent and primate species, whereas the last common ancestor of humans and macaque monkeys dates back only 25 million years (Kumar and Hedges, 1998). Unlike the human in which the neocortex represents 80% of the brain, only 28% of the rat brain is neocortex. In the macaque it encompasses 72% of the brain volume (Passingham, 2009). The lack of morphological diversity between individual rats that allows for better localization and registration is partially owing to the smooth cortex that lacks the extensive folding seen across primate species. Functionally, there are many areas in the primate brain that do not exist in the brains of rats such as subdivisions of the prefrontal cortex including granular prefrontal regions (Preuss, 1995; cf. Uylings et al., 2003) or the extensive functional parcellation of visual areas (Uylings et al., 2003). The decreased surface area, functional specialization, and overall complexity likely result in the dramatically different behavioral repertoires and cognitive abilities that further limit the rat's applicability to characterize more advanced human brain function and organization. Routine perceptual and cognitive tasks commonly utilized in behavioral and electrophysiological studies of macaques, beyond paradigms of emotional learning (LeDoux, 2003) and spatial memory (Barnes, 1988), are typically not possible in rats (cf. Kesner and Churchwell, 2011).

Most relevant to this review, the differences of rodent and primate brains are also reflected in their RSN topology. As will be discussed in the next section, the most commonly reported higher-order human RSNs are more spatially distributed, thereby demonstrating synchronization beyond bilateral homologs and including other non-contiguous areas throughout the brain. Seed region analysis has revealed more distributed networks in the rat though these are typically much “noisier” and do not share the same level of robustness and reproducibility as observed in the distributed networks seen in both humans and macaques. Liang and colleagues (2011) have applied graph metrics to examine inter-RSN topology, revealing a broad grouping of the rat RSNs into three clusters/modules. The organization of the RSNs into modules, however, is still a far departure from the distributed within-network connectivity seen in primate species (a more distributed, default-mode like network has been recently reported in rats, see below). RSNs of humans and macaques are much more spatially distributed across cortical and subcortical areas (Beckmann et al., 2005; Damoiseaux et al., 2006; Vincent et al., 2007; Hutchison et al., 2011). Even the aforementioned bilateral sensory and motor networks tend to encompass larger extents of cortex and in humans, multiple discrete functional, and anatomical areas. Many task-based studies of rats also do not report networks beyond uni- or bilateral activation of specific areas though there have been reports of more distributed task- and electrically-evoked networks (Zhao et al., 2008). The weaker synchronization across distributed structures in the rat brain could be a reflection of limited ongoing “higher-order” processing such as spontaneous cognition or predictive processing, features that may represent a manifestation of a more evolved form of network topology. It is for these reasons that research on other primate species; in particular macaque monkeys that have been used as surrogates for the study of human brain function for several decades will be essential for exploring more complex questions surrounding RS-fMRI investigations.

### Homologous macaque networks

Given that monkeys have been shown to share features of behavior and cognition observed in humans, it would seem likely that they also share similar organization of functional brain networks. Exploratory techniques such as independent component analysis (ICA) have consistently revealed a set of core RSNs in the human (Beckmann et al., 2005; Damoiseaux et al., 2006; De Luca et al., 2006; Smith et al., 2009). The RSNs reflect functional systems supporting core perceptual and cognitive processes. Application of the same methodology has revealed potential homologs across many of these networks in the macaque. Figures 1 and 2 display selected RSNs of the human (*N* = 12; data from Hutchison et al., 2012) and isoflurane-anesthetized macaques (Hutchison et al., 2011) reveled from ICA (model order = 20) of resting-state data. The networks carry putative labels based on their close correspondence with task-based networks and include the “somatomotor,” “visual,” “ventral premotor,” “auditory,” “higher-order visual” encompassing extra-striate visual areas, “executive,” “default-mode” (DMN), and attention/oculomotor networks. Note that although the functional networks are displayed on inflated cortices, the intra-network connectivity is not restricted to cortical areas and RSNs display functional connectivity patterns with specific thalamic (Zhang et al., 2010a) and cerebellar nuclei (Krienen and Buckner, 2009).

#### Default-mode network

The most commonly investigated and perhaps most controversial network is the default-mode network (DMN). In humans, it bilaterally encompasses the posterior cingulate (PCC)/retosplenial cortex (Rsp)/precuneus (PGm), ventral and dorsal medial prefrontal cortex, inferior parietal lobule, lateral temporal cortex, and hippocampal formation (see Figure 2). The DMN reduces its activity during goal-directed behavior and has been implicated in a range of functions including self-referential thought, both internal and external monitoring, memory consolidation, supporting consciousness, and daydreaming, among others (Mason et al., 2007; Raichle and Snyder, 2007; Buckner et al., 2008). Given its potential role in oft-labeled “human” processes, assessing its presence in other species is of great interest.

Most resting-state investigations using seed-based or ICA approaches have not reported a potential homolog for the DMN in the rat. Recently, however, two separate reports have supported a DMN-like network in the awake and anesthetized rat brain (Upadhyay et al., 2011; Lu et al., 2012) converging on a similar, though not identical topology patterns. These results raise a number of interesting questions. Could this network represent a precursor to the primate DMN? What is its functional role? Before these questions can be addressed it will first be essential to determine the validity of these findings. It is unclear why previous reports did not reveal the same network when using ICA at multiple model orders at single and group levels. As mentioned, a distributed network at rest is also not typical of most reported RSNs in rat investigations. Finally, by definition, the DMN activity should decrease during task performance and this functional role will have to be addressed in a similar manner as Mantini et al. (2011).

**Figure 3:**
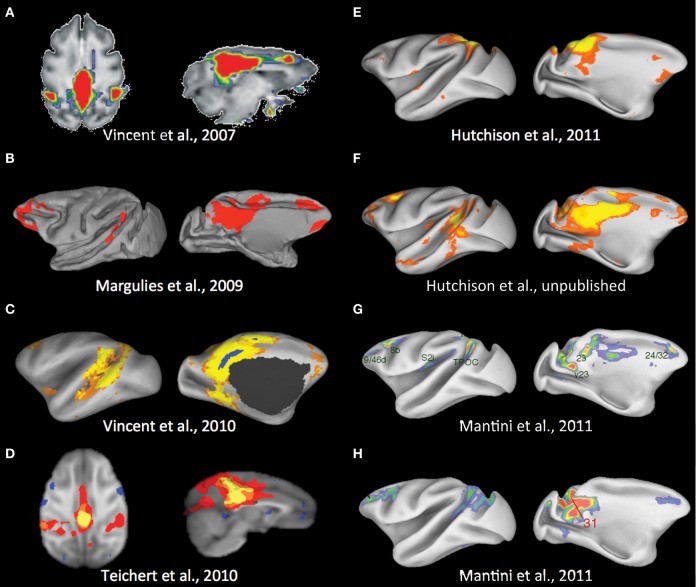
**Potential default-mode network homolog of the macaque across multiple studies.** See text for description. Modified with permission from **(A)** Vincent et al. (2007); **(B)** Margulies et al. (2009); **(C)** Vincent et al. (2010); **(D)** Teichert et al. (2010); **(E)** Hutchison et al. (2011); **(F)** Unpublished results from the same data set as Hutchison et al. (2011); **(G,H)** Mantini et al. (2011).

The identification of a DMN in non-human primates also remains ambiguous, though a consensus is beginning to emerge (Figure 3). Vincent and colleagues (2007) first reported a potential candidate for a homologous macaque DMN (Figure 3A). An anatomically placed seed in the posterior midline encompassing areas of the PCC (areas 23 and 31) and a portion of the PGm (area 7m) of isoflurane anesthetized macaques was found to be functionally connected with lateral temporoparietal cortex (including area 7a and superior temporal gyrus) and posterior parahippocampal cortex (PPHC). There were also strong correlations with the dorsal medial prefrontal cortex (dmPFC; area 9) though there is considerable overlap with the anterior cingulate cortex (ACC) (area 24c). Using the same dataset, Margulies and colleagues showed that the heterogeneous features of the posteromedial cortex could be revealed using RS-fMRI and that connectivity profiles are greatly dependent upon the selection of the seed region within its individual subunits (Rsp, PCC, PGm) (Margulies et al., 2009). Seeds restricted to the PCC (area 23/31) most closely resembled that of the previous study and included characteristic human DMN nodes (Figure 3B). There were however, notable differences as the ventral medial PFC (vmPFC; areas 10m, 32, and 14r), dorsolateral prefrontal cortex (dlPFC), and inferior parietal lobule were functionally connected to the PCC seed whereas lateral temporoparietal cortex and hippocampal formation connectivity were absent. Their results also advanced the idea that the PGm is not in fact a component of the DMN (Buckner et al., 2008). A third study of the same isoflurane anesthetized monkeys using a PCC/Rsp seed defined from a PPHC connectivity map revealed a combination of areas from the previous studies albeit with limited dlPFC connectivity (Vincent et al., 2010; Figure 3C). A separate seed-region based investigation of three isoflurane anesthetized macaques did not corroborate the potential homologous cortical areas as medial frontal, dorsal frontal, and hippocampal regions were absent when using a posteromedial cortex seed (Teichert et al., 2010; Figure 3D). Given the dependency on the seed location shown in the aforementioned studies—a finding that has been observed in humans (Cole et al., 2010)—it is possible the seed encompassed a large proportion of the PGm.

To avoid the constraints of seed selection, we followed the approach of many human investigations and implemented ICA with a low model order (Hutchison et al., 2011). The closest qualitative component matching a potential DMN homolog contained the PGm with some extension into the PCC, areas PG and PE of the parietal cortex, and unilateral vmPFC (area 14r and 10m) and ACC area 24a/c (Figure 3E). Given the previous results and the significant portions of the DMN architecture that are missing, this more likely reflects a combination of the PGm sensorimotor and cognitive networks (Margulies et al., 2009). To rule out issues with the quality of our data or confound variables, we have since placed a midline spherical seed (radius = 1.5 mm) in the PCC (area 23/31). The results show homologous areas across all nodes of the human DMN (Hutchison et al., unpublished data; Figure 3F) and taken with the other studies most likely represents the full DMN homolog of the macaque. Future investigation and application of ICA will be necessary to explain why this DMN component does not emerge in either group or single subject ICA (Moeller et al., 2009; Hutchison et al., 2011) as it is very robustly identified in human studies.

Most recently, a meta-analysis of fMRI data collected from ten awake monkeys performing tasks showed a network of regions that decreased in activity when the task demands shifted from a passive task to externally oriented processing (Mantini et al., 2011)—a defining feature of the human DMN. The network included medial, cingulate, parietal, and prefrontal regions (Figure 3G) that demonstrate substantial spatial overlap with our PCC seed-based network with the exclusion of lateral temporoparietal cortex and the hippocampal formation. The findings further corroborate this RSN as the monkey equivalent of the human DMN. It is important to consider that within the same paper, however, seeding areas within nodes of the network in awake fixating monkeys (*N* = 4) did not reproduce the identical network, though seeding area 31 produced the closest qualitative match (Figure 3H). Taken together, there does seem to be a general consensus as to a homologous DMN and its components in the macaque that can be reveled with resting-state approaches. For it to become a dependent variable in experimental manipulations, future work examining both physiological and methodological variables will be needed to explain the lack of robustness within and across studies.

#### Fronto-parietal networks

Apparent in Figure 2 (bottom row) is the presence of lateralized, fronto-parietal networks in the human whose potential homolog in the macaque is symmetric. The networks have been implicated in cognitive attentional and oculomotor processes as well as memory and language functions (Beckmann et al., 2005; Jafri et al., 2008; Smith et al., 2009). We further explored the fronto-parietal connectivity of both species in a seed-region analysis of the same human and monkey datasets (Hutchison et al., 2012). As expected, there were consistent ipsilateral functional connections of the frontal eye fields (FEF) with fronto-parietal cortical areas across both species. These included the intraparietal sulcus, dlPFC, ACC, and supplementary eye fields. The analysis also revealed greater lateralization of connectivity with the FEF in both hemispheres in humans than in monkeys, corroborating the findings of the ICA. We suggest that the asymmetry in correlation patterns reflect a real functional difference that is consistent with the general evolution to increased functional specialization and lateralization in humans.

**Figure 4:**
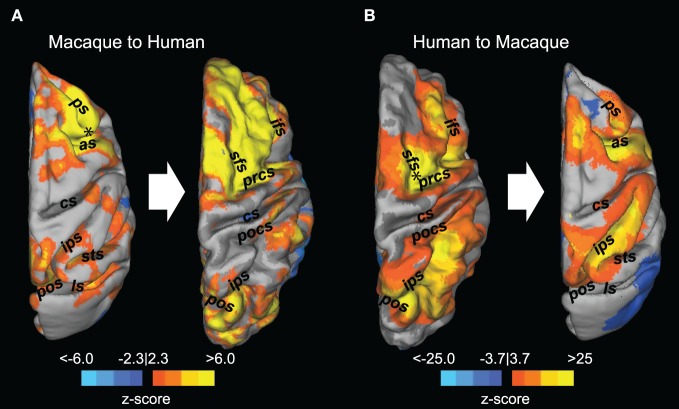
**Registration of resting-state fronto-parietal functional connectivity maps between macaques and humans.** Thresholded z-score maps derived from a seed-based analysis using a seed placed in the right frontal eye field (black asterisk) are superimposed on the dorsal view of the macaque (**A**, left) and human (**B**, left) cortical surface. The connectivity maps were then transformed into the space of the other species using cortical surface-based transformation (**A,B** right side). as, arcuate sulcus; cs, central sulcus; ifs, inferior frontal sulcus; ls, lateral sulcus; pos, parieto-occipital sulcus; pocs, posterior central sulcus; prcs, precentral sulcus; ps, principal sulcus; sfs, superior frontal sulcus; sts, superior temporal sulcus. Reprinted with permission from Hutchison et al. (2012).

Owing to the substantial differences in overall brain size as well as organization of different cytoarchitectonic areas, there can be difficulties identifying common spatial topologies between species. To aid in comparison of the intrahemisphere connectivity, we employed surface-based mapping based upon previously identified homologous landmarks (Van Essen, 2004; Denys et al., 2004; Orban et al., 2004; Van Essen and Dierker, 2007) that serves to non-linearly transform the macaque connectivity maps into the format of the human brain and vice versa. The use of cortical surface-based transformation of connectivity maps between species further corroborated the remarkable ispilateral organization of the FEF functional connectivity (Figure 4). Overall, the results indicate an evolutionarily preserved fronto-parietal system, but also present the opportunity to investigate the evolutionary predecessor of the lateralized, human networks.

#### Detailed mapping

**Figure 5:**
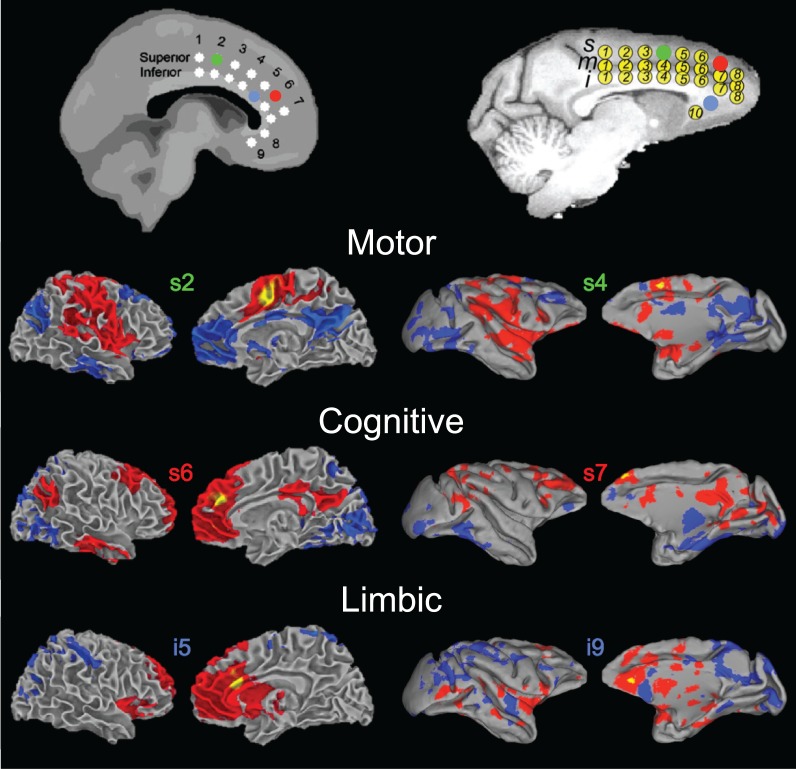
**Homologous functional subdivisions of the anterior cingulate cortex.** Functional connectivity profiles of seed regions within the anterior cingulate cortex are shown for the human (left column) and macaque (right column). Color-coded seed locations are shown are shown on standard brain templates for the humans (MNI) and monkeys (F99), respectively (top). Putative functional roles are labeled. Modified with permission from Margulies et al. (2007); Hutchison et al. (2012a).

It is also important to recognize that the primary RSNs do not represent the extent of large-scale networks in the human or non-human primate brain. Unique connectivity profiles have been reported when using both hypothesis driven (Krienen and Buckner, 2009; Vincent et al., 2010; Mars et al., 2011) and exploratory (Damoiseaux et al., 2006; Liao et al., 2009; Moeller et al., 2009; Hutchison et al., 2011) techniques. RS-fMRI offers an excellent opportunity to examine functional subunits or parcellation within structures. As mentioned above, distinct patterns of functional connectivity were demonstrated within the posteromedial cortex or both species, with each subdivision suggesting a discrete functional role (Margulies et al., 2009). A similar analysis procedure has been used to delineate subdivisions of the ACC. Margulies and coworkers (2007) placed spherical seeds throughout the caudal, rostral, and subgenual ACC in human subjects (Figure 5, left panel). They found that posterior seeds were positively correlated with cortical motor-related areas and anti-correlated with posterior and subgenual cingulate. More anterior seeds showed strong positive correlations with PPC and dlPFC. The ventral ACC was positively correlated with insular cortex. These functional connectivity patterns were consistent with the popular model that proposes a distinction between a dorsal cognitive and a ventral affective ACC subdivision (Bush et al., 2000). Recently, we performed a similar analysis in macaque monkeys (Hutchison et al., 2012a). We found that the functional connectivity of the ACC varied systematically along the rostral/caudal and dorsal/ventral axis, thereby confirming previous anatomical tracer and lesion studies in monkeys (Pandya et al., 1981; Vogt et al., 1987; Barbas et al., 1999; Rudebeck et al., 2006). We were able to delineate several subdivisions and identified separate primary networks within the ACC. The functional connectivity maps of individual seeds showed a remarkable similarity with those found by Margulies et al. (2007) in humans. In particular, we were able to identify macaque ACC seeds that corresponded to the motor, cognitive, and limbic subdivisions (Figure 5, right panel). These two studies demonstrate that RS-fMRI is a useful tool for comparative mapping of brain networks in humans and non-human macaques.

### Comparative conclusions

Functional networks allow brain areas to work in concert to support the diverse range of cognitive processes necessary for the selection and implementation of appropriate behaviors. This is essential for the survival and reproductive success of the individual and by extension, the species. Therefore, the brain topology of a species is the product of evolutionary changes driven by a diverse range of internal and external selection pressures. Universal properties commonly emerge such as maximizing high efficiency of information transfer while maintaining low physical connection cost, small-world properties found across multiple species (Bullmore and Sporns, 2009; Sporns, 2010). That is not to say that all brains are organized in the same fashion. The specific development and arrangement of neurons across multiple scales vary greatly between species that are not simply a product of increased size. RS-fMRI is well suited for the examination of the evolution of brain networks and evaluation of homologies between species. Recent work has suggested that RSNs and their reciprocal temporal features are ubiquitous across mammalian species. The results also show that as brain complexity increases, bringing with it a more diverse repertoire of cognitive and behavioral states, new RSN topology develops. Interhemispheric communication seems to represent the most fundamental large-scale network structure. More sentient species such as the macaque and human show distributed networks encompassing multiple brain areas beyond contralateral homologs. Hierarchical evaluation of such networks does, however, show that the homologous structures are most strongly connected and can represent subsystems within the larger network. Presumably these networks, whose cost in terms of wiring and development is high, evolved to facilitate greater information integration and computation. At the highest complexity level, humans demonstrate robust lateralization of the distributed fronto-parietal RSNs, a property not found in non-human primate species and could suggest that this specialization is a further adaption necessary for human-specific behaviors such as language. From an applied research perspective, the findings support the use of non-human primates as a suitable animal model for the study of network disruptions in disease and exploration of the true underpinnings of the RS-fMRI signal.

## Correlates of spontaneous bold activity and RSNs

The BOLD signal is not a direct measure of neural activity as would be recorded in electrophysiological experiments. Instead, it represents a surrogate signal reflecting local variations in the deoxyhemoglobin concentration that reflects fluctuations in blood flow, blood volume, and oxygen metabolism that are then partially coupled to the underlying neural activity (Raichle and Mintun, 2006). Task-evoked BOLD responses have been best linked to the induced changes in local field potentials (LFPs; Logothetis et al., 2001) and mechanisms of neurovascular coupling have been proposed (Attwell and Iadecola, 2002; Uludağ et al., 2004; Raichle and Mintun, 2006; Iadecola and Nedergaard, 2007). The same correlation has not been confirmed for spontaneous hemodynamic fluctuations and it is yet to be determined if the coupling mechanisms are the same across spontaneous and evoked states.

There are a number of studies employing simultaneous electroencephalography (EEG)-fMRI in humans that have explored the relation of spatial and temporal hemodynamic patterns to bands of electrophysiological activity (Goldman et al., 2002; Laufs et al., 2003; Mantini et al., 2007, 2011; Sammer et al., 2007; Laufs, 2008, 2010; He et al., 2008; Olbrich et al., 2009; Britz et al., 2010; Michels et al., 2010; Wu et al., 2010; Musso et al., 2010). Many of these studies have shown that specific frequency bandwidths (or power fluctuations of the bandwidth) are in fact correlated with the ongoing hemodynamic fluctuations. Though these results do much to support a neural origin of the RS-fMRI signal, drawing overall conclusions from the studies is difficult as they each implicate different bands across the frequency spectrum including delta, theta, alpha, beta, and gamma. Other evidence has begun to reconcile these findings by ascribing unique electrophysiological signatures to different brain states and RSNs that are represented by power variations across the EEG range (Laufs et al., 2003; Mantini et al., 2007; Britz et al., 2010; Musso et al., 2010; for review see Laufs, 2008).

First proposed by Fox and Raichle (2007), infraslow oscillations represent another potential candidate. Using direct current-coupled EEG, which circumvents the limited recording bandwidth of most EEG systems (>0.5 Hz), large-scale infraslow oscillations (0.02–0.2 Hz) can be recorded across widespread regions in the human cortex (Vanhatalo et al., 2004). The oscillations are themselves correlated with changes in the power of higher frequency bands including gamma, leading to the notion of a causal role between the two processes in which the infraslow oscillations modulate the power of higher frequency activity. In this model, the rapid fluctuations coordinate the neuronal activity at small spatial scales, whereas the much slower power fluctuations allow for long-range coordination. This is supported by empirical evidence (Buzsáki and Draguhn, 2004), however its direct relationship to BOLD fluctuations remains to be determined.

The failure to record the complete range of physiologically relevant electrophysiological signals when using standard EEG highlights one of a number of its disadvantages that prevent adequate exploration and understanding of the neural activity underlying hemodynamic fluctuations and their organization into complex networks. The most prominent is that EEG, despite its excellent temporal resolution, is unable to accurately discern or record all cortical and subcortical activity (Gloor, 1985). The limited source localization is due to biophysical challenges related to convolution of the cortical surface, distortion from cerebral spinal fluid, neuron orientation, synchronization, skull conduction, and other sources of attenuation or loss (Ritter and Villringer, 2006) that further exacerbate the inverse problem. The problem refers to the infinite number of possible locations and magnitudes of the intracranial current sources that make reconstructing a unique mathematical solution, and by consequence a precise spatial mapping, impossible (Niedermeyer and da Silva, 2004). Electrocorticography (ECoG) can circumvent several of these limitations and allow better source localization, but it is extremely invasive and only suitable for a small group of patient populations. He et al. (2008) have reported that a similar “correlation structure” of the sensorimotor network recorded by ECoG and RS-fMRI in patients undergoing surgical treatment for intractable epilepsy. Slow cortical potentials (<0.5 Hz) and gamma frequency power were found to best correspond with the RS-BOLD fluctuation profiles across multiple states (He et al., 2008).

Beyond the electrophysiological correlates, a number of other hemodynamic and metabolic variables have been put forth by Fox and Raichle (2007) and require consideration. Oxygen availability, nicotinamide adenine dinucleotide levels, spontaneous neurotransmitter release, cytochrome oxidase activity, blood volume, and blood flow demonstrate spontaneous low-frequency fluctuations that can have 1/*f* distributions and similar spatial patterns to those seen with BOLD (Fox and Raichle, 2007). Therefore, precise spatial localization and characterization of temporal patterns must come from recording electrodes and optical probes placed within the brain. Ideally, these will be recorded simultaneously with RS-fMRI. Nir and colleagues (2008) have reported slow (<0.1 Hz) spontaneous fluctuations of neuronal activity (LFP gamma power modulations) in the auditory cortex of a small group of awake patients as well as significant interhemispheric correlations between the homologous areas using bilateral single-unit and LFP recordings (Nir et al., 2008). However, owing to the same restrictions requiring the examination of non-human primates to elucidate the task-based neurovascular coupling, non-human primates will be critical in establishing the correlate of the low frequency oscillations as distributed areas can be directly assessed with depth electrodes and optical probes, high-field studies, and pharmacological investigations.

The growing body of animal studies does support the notion that resting-state BOLD fluctuations of cortical and sub-cortical regions originate from the coupling of spontaneous neuronal activity to a hemodynamic response function. Early multi-modal evidence in anesthetized rats demonstrated tight coupling between spontaneous cerebrovascular fluctuations and bursts of electrocortical activity (Golanov et al., 1994). Pioneering work in the macaque has further established slow fluctuations of power in the gamma frequency range as the leading candidate for the neural correlate of spontaneous BOLD fluctuations (Leopold et al., 2003; Shmuel and Leopold, 2008). Leopold and colleagues (2003) were able to show that the power of gamma at a particular moment fluctuates, albeit at a much slower rate (<0.1 Hz) than its LFP activity. Besides sharing a similar frequency as the hemodynamic changes, the slow power fluctuations exhibited 1/*f* behavior and was correlated across large regions of cortex (Leopold et al., 2003). Later, it was shown using simultaneous fMRI and LFP recordings again in non-human primates, that the power fluctuations are in fact directly correlated with spontaneous BOLD fluctuations (Shmuel and Leopold, 2008). Macaques have also been used to support a neural origin of the global component of RS-fMRI (Schölvinck et al., 2010) and investigate differences of electrically induced and spontaneous activity (Matsui et al., 2011).

Non-human primates also afford the opportunity to invasively explore the generalizability of the neurovascular mechanisms contributing to the spontaneous BOLD fluctuations across cortical and subcortical areas. Implicit in most hypothesis-driven (seed-region analysis) and exploratory (ICA, principal component analysis, clustering) approaches is an assumption of regional homogeneity in the mechanisms underlying neurovascular coupling. However, recent task-based fMRI investigations of the rat (Sloan et al., 2010) and humans (Martuzzi et al., 2009; Conner et al., 2011; Gonzalez-Castillo et al., 2012) examining distributed response patterns have revealed variations in regional coupling between neural activity and metabolism. Whereas task-based studies may constrain their analysis to the task paradigm, increase averaging, and utilize more flexible or dynamic hemodynamic response functions (Gonzalez-Castillo et al., 2012), RS-fMRI does not afford such time-locked events and are dependent upon comparing the timecourses of voxels containing considerable differences in cell type, laminar organization, connectivity, in addition to variations in vascular, neuron, synapse, and astrocyte density (Logothetis and Wandell, 2004). Detailed optical imaging in primate studies will help elucidate the mechanisms and scale of the variability and could suggest that measures beyond region correlations will be necessary to truly characterize ongoing functional relationships that may be captured using RS-fMRI.

## Anesthesia and states

A large proportion of the resting-state investigations of animals have utilized anesthesia as a method to eliminate motion effects, physiological stress, and training requirements. As outlined throughout this review, RSNs have been found in rodents and macaques despite the use of various types of anesthesia. In rodents, these have included alpha-chloralose (Lu et al., 2007; Majeed et al., 2009), medotomidine (Pawela et al., 2008, 2010; Zhao et al., 2008; Magnuson et al., 2010), ketamine/xylazine (Hutchison et al., 2010), and isoflurane (Kannurpatti et al., 2008; Hutchison et al., 2010; van Meer et al., 2010; Liu et al., 2011; Wang et al., 2011). Isoflurane represents the most commonly used anesthetic in monkeys (Vincent et al., 2007; Shmuel and Leopold, 2008; Teichert et al., 2010; Hutchison et al., 2011; Mars et al., 2011) though propofol (Matsui et al., 2011; Adachi et al., 2012) and a combination of ketamine and medetomidine (Moeller et al., 2009) have been used successfully. Despite convergence of results across anesthesia types and even with awake animals (Zhang et al., 2010b; Liang et al., 2011; Mantini et al., 2011) it is incorrect to say that quantifiable differences do not exist between the different states (Lu et al., 2007; Moeller et al., 2009; Williams et al., 2010; Wang et al., 2011). The mechanisms of action of many anesthetics remain poorly understood, but certainly modulate neural activity and consequently influence the cerebral blood flow if not effecting blood flow directly. Isoflurane in particular has been shown to disrupt functional thalamocortical connectivity (Alkire et al., 2000; Steriade, 2001; Arhem et al., 2003) in addition to causing vasodilation that can alter cerebrovascular activity (Farber et al., 1997; White and Alkire, 2003; Schlünzen et al., 2006). The effects could be manifested as changes in the correlation strength, localization, or inclusion of distributed nodes within specific networks (Lu et al., 2007; Vincent et al., 2007; Liu et al., 2011; Wang et al., 2011). Anesthesia can also limit longitudinal studies in cases such as alpha-chloralose that require animal sacrifice or recoverable anesthetics that require a necessary interval between scanning sessions. Finally, there are concerns of possible drug interactions when studying pharmacological interventions.

Despite the disadvantages and limitations, the utility of anesthesia should not be understated. Beyond allowing extended, motion-free acquisition in naive animals, anesthesia experimentation can serve to explore the fundamental physiological relationships underlying spontaneous fluctuations and functional connectivity by exploiting their unique mechanisms of action and effect on neural activity, neurovascular coupling, and vascular reactivity. Further, anesthesia can eliminate conscious processes such as passive mind wandering, active monitoring, memory formation, or changes in attention and arousal during image acquisition that may confound certain experiments (Hutchison et al., 2012b). Reciprocally, there is the potential to explore the mechanisms that account for the anesthesia's diverse effects on memory, pain, and consciousness. While the use of anesthesia in human research is possible (Greicius et al., 2008) it is severely limited and provides an additional need for animal investigations.

## Relationship between function and structure

There are a number of fundamental questions regarding the potential relationship between functional connectivity, measured as the temporal relationships between brain regions, and the underlying structural connectivity that represents the anatomical white matter fiber tracts that still remain unanswered. By definition, RSNs are composed of anatomically separated brain regions. These can include contralateral homologs or more distributed patterns within and between hemispheres. Given the growing evidence supporting a neural origin of resting-state fluctuations and their synchronization, many have hypothesized that the functional connectivity is supported by direct structural connections (Damoiseaux and Greicius, 2009). While direct evidence is limited in human subjects, simulated and empirical investigations of humans have suggested an overall good correspondence between the two (Damoiseaux and Greicius, 2009; Greicius et al., 2009), although clear discrepancies have emerged.

The majority of studies examining the relationship between spontaneous BOLD correlations and anatomical connectivity have used diffusion imaging techniques such as diffusion tensor imaging (DTI) or diffusion spectrum imaging (DSI). At a local level, Koch and coworkers found that regions on either side of a sulcus showing high functionally connectivity were also structurally connected by short-range fibers (Koch et al., 2002). This has since been shown at the whole brain level, in which regions with a higher level of structural connectivity showed higher levels of functional connectivity (Honey et al., 2007, 2009; Hagmann et al., 2008). Indeed, almost all functionally linked regions of the most commonly reported RSNs appear to be roughly constrained by known white matter tracts (Honey et al., 2007; Vincent et al., 2007; Greicius et al., 2009; van den Heuvel et al., 2009). The relationship between structural and functional connections is not, however, one-to-one and there are a number of discrepancies. Studies have reported areas that share no direct connections (Habas, 2009; Honey et al., 2009; Krienen and Buckner, 2009). For example, primary visual cortex has been found to be robustly connected to its contralateral homolog, though no direct connections exist (Van Essen et al., 1982). This implies that some of the reported functional connectivity is driven by polysynaptic pathways. The opposite pattern has also been reported in which areas known to have structural connections do not show functional connectivity (Adachi et al., 2012; Hutchison et al., 2012). Evaluation of the correspondence between connectivity types is made difficult by methodological limitations of diffusion techniques that do not allow precise delineation of the origins, crossings, and terminations of pathways, thereby restricting the interpretation of results. The few case studies examining congenital or surgical alteration of the callosal fiber connections have produced mixed results (Quigley et al., 2003; Johnston et al., 2008; Uddin et al., 2008; Tyszka et al., 2011). Further insight into the relationship between the two connectivity types is needed and will likely come from an experimental system in which anatomical connectivity can be more easily assessed and manipulated—that is, in an animal model. Histological dissection and staining, degeneration methods, and axonal tracing carried out in non-human primates remains the gold standard for uncovering the precise information concerning the origins and terminations of white matter connections.

The first qualitative comparisons of functional resting-state connectivity maps and structural connectivity maps derived from experimental tracer studies in the macaque demonstrated remarkable consistency between the patterns (Vincent et al., 2007; Margulies et al., 2009; Mars et al., 2011, Figure 6). In fact, macaque tracing findings can be accurately extrapolated to predict human functional connectivity patterns (Margulies et al., 2009; Kelly et al., 2010).

**Figure 6:**
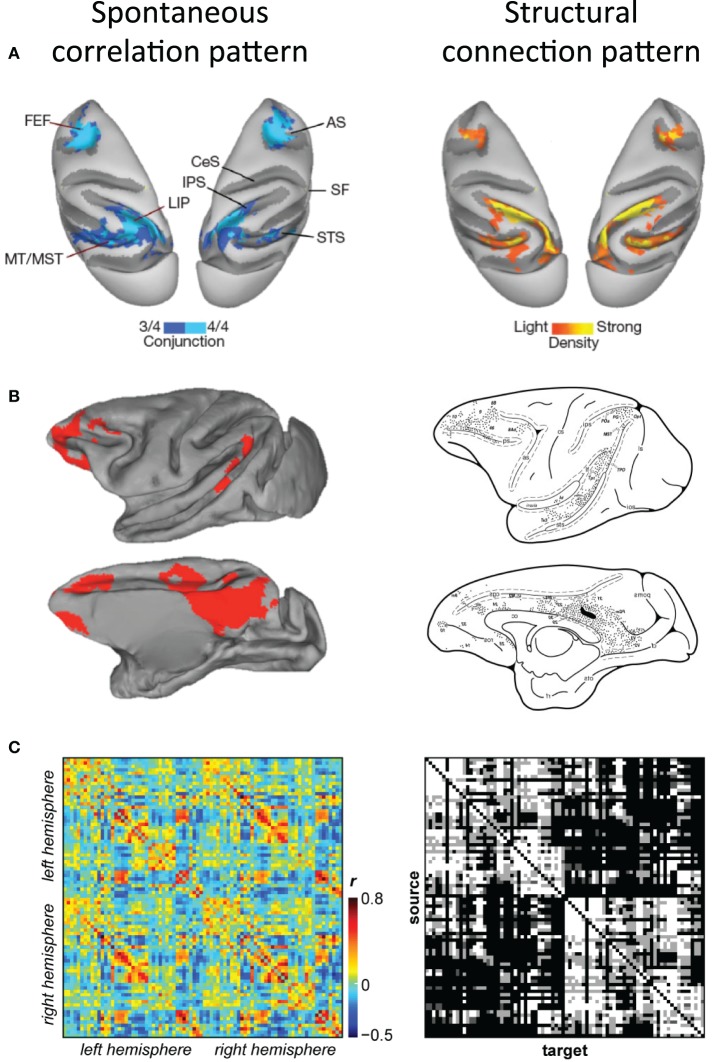
**Correspondence of functional and structural connectivity patterns in the macaque. (A)** Conjunction map of functional connectivity the macaque oculomotor system (left) and density of cells labeled by retrograde tracer injections into right LIP (right) displayed on dorsal views of both hemispheres. **(B)** Correlation map of functional connectivity of macaque posterior cingulate cortex (left) and the structural connectivity patterns injection of tracers within a location comparable to the respective seed region (right) displayed on medial and lateral views of the left hemisphere. **(C)** Functional connectivity matrix of 82 cortical seed regions averaged across six macaques (left) and the corresponding structural connectivity matrix derived from the CoCoMac database (right). Modified with permission from **(A)** Vincent et al. (2007); **(B)** Margulies et al. (2009) and Morecraft et al. (2004); **(C)** Shen et al., in preparation.

To allow for a more quantitative assessment of functional/structural relationships it is necessary to examine the correspondence beyond single areas. Recent work has compared macaque RS-fMRI connectivity to structural connectivity derived from macaque axonal tract tracing studies contained within the CoCoMac database (Adachi et al., 2012). CoCoMac is a systematic record of the known anatomical connectivity of the primate brain containing details of hundreds of tracing studies in their original descriptions (Stephan et al., 2001). The primary finding of this work was that functional connectivity between areas with no direct structural connection is driven by common afferents and common efferents (as opposed to serial relays). The work however, did not attempt to explore the overall correspondence between the connectivity types and was limited to unilateral visual and sensorimotor areas in two monkeys. Current investigations are building on this work, establishing similarity measures between functional and structural connectivity across the complete extent of both cortical hemispheres (Shen et al., in preparation; Figure 6C). The results revealed a relatively high degree of overlap between connectivity measures. Further, the strength of functional connectivity was proportional to the strength of the anatomical connection. These findings confirm the general notion that white matter constrains functional architecture, however, as has been previously reported, the relationship between the two is not perfect. Future investigations directly comparing diffusion tractography and functional connectivity results with neuroanatomical tracing data of the same monkey will be necessary to most accurately determine the precise relationship of functional and structural connectivity in the primate.

The macaque model has also allowed for the controlled evaluation of fiber pathway contribution to functional connectivity through surgical manipulation. As stated, there are inconsistent results when examining patients with agenesis or resection of the corpus callosum. A case study of a 6-year-old child following resection of the corpus callosum (Johnston et al., 2008) and of a small group (*N* = 3) of patients with agenesis (Quigley et al., 2003) found significantly decreased functional connectivity between the neocortices. However, contradictory reports from a patient (age = 73) who underwent complete forebrain commissurotomy (Uddin et al., 2008) and a sample (*N* = 8) of patients with complete agenesis of the corpus callosum (Tyszka et al., 2011) found preserved bilateral connectivity. In response to these discrepancies, that may be possibly related to compensatory mechanisms occurring over time, Croxson et al. (2011) scanned monkeys (*N* = 4) before and after surgical transection of the forebrain commissures (including the body and genu of the corpus callosum, anterior commissure, and splenium including the hippocampal commissure). The authors found significantly decreased interhemispheric correlations between pre- and post- operative scans across multiple bilateral homologs.

Overall, the strong positive similarity values between functional and structural connectivity matrices and the loss of interhemispheric correlations following surgical resection of the tracts suggest that the two measures are intricately related. The relationship, however, is not one-to-one as evinced by a less than perfect correlation and presence of correlations in the absence of direct connections. The results are not necessarily surprising given that the structural brain network needs to facilitate a vast array of functional configurations to achieve different states (van den Heuvel and Hulshoff Pol, 2010). It is here that the non-human primate will continue to serve an invaluable role by allowing the exploration of polysynaptic connectivity and regulation by common inputs.

## RSN and disease

Networks facilitate efficient information transfer and allow the emergence of properties not possible when the nodes are in isolation. These could include, for example, increased processing capabilities, stability, or resource sharing. However, alteration or breakdown of the network, especially of central (hub) nodes, can create detrimental dynamics and catastrophic failure across the entire system. The brain is especially sensitive to manipulations that alter its functional and structural organization. A growing and promising avenue of research is exploring the use of RS-fMRI measures in assessing clinical disorders; the overarching hypothesis across many of these studies being that alteration of brain networks are the cause or consequence of the abnormal manifestations characteristic of the disease. The technique is particularly well suited for investigations of non-normal populations, such as subjects with severe cognitive or physical impairments compared to other methodologies, including task-based fMRI. This is because resting-state investigations require minimal task compliance and therefore allow for accurate comparisons of brain connectivity and dynamics. For example, a task requiring memory encoding can be of particular concern when evaluating patients suffering from neurodegenerative diseases. Caution, however, must be taken when comparing groups that may exhibit systematic differences unrelated to paradigm such as motion that can induce systematic, but spurious correlation structures throughout the brain (Power et al., 2012).

Although their meaning is not fully understood, changes in functional RSNs have been reported in multiple psychiatric and neurologic disorders including depression (Greicius et al., 2007; Kühn and Gallinat, 2011; Lui et al., 2011), attention deficit-hyperactivity disorder (Castellanos et al., 2008; Fair et al., 2010), schizophrenia (Whitfield-Gabrieli et al., 2009; Kühn and Gallinat, 2011; Bassett et al., 2012), Alzheimer's disease (Greicius et al., 2004; Chen et al., 2011), epilepsy (Waites et al., 2006; Zhang et al., 2011), disorders of consciousness (Soddu et al., 2011) including coma (Norton et al., in press), multiple sclerosis (Lowe et al., 2002, 2008), and amyotrophic lateral sclerosis (Mohammadi et al., 2009) (for reviews see Auer, 2008; Greicius, 2008; van den Heuvel and Hulshoff Pol, 2010). Many early studies focused on the default-mode RSN, as the network seems particularly sensitive to disruption in disease states, but more recent work has now started to examine other networks as well as changes in the overall organization of functional brain network using graph analysis techniques (Jafri et al., 2008). For example, through graph analysis of resting-state data it was revealed that the locations of high concentrations of amyloid deposits in Alzheimer's disease patients were highly correlated with the location of highly connected hub-regions in the human brain suggesting that disruption of integrative hubs may result in the decreased functional brain efficiency in these patients (Buckner et al., 2008). Taken together, the extensive documentation of altered RSN topology suggests that brain diseases are targeting interconnected cortical networks, rather than a single region and may help explain some of the complex manifestations seen in these patient populations.

Given that the examination of spatiotemporal properties of RSNs studied with RS-fMRI can delineate abnormal neural functional architecture, the natural extension of the methodology would be using RSN-related metrics as potential screening devices for disease. However, many of the robust changes across the range of aforementioned disorders have been derived and significance-tested for “proof of concept” at the group level. These represent valuable contributions toward understanding abnormal brain activity and connectivity, but characterizing patterns of functional variability between normal and patient groups is far from providing clinical diagnostics at the single-subject level. The correlative results also present a directionality problem, in that the relationship between the disease and altered connectivity are unclear. The functional disruptions could represent a consequence of the disease or be the underlying cause and this could vary across disease types. Given the extraordinary potential for RSNs as possible diagnostic or prognostic markers, it is crucial to understand the mechanisms by which they are altered. While rodent models do share features of brain organization and afford examination of genetically altered models, the homologous spatial and temporal brain properties establish the macaque monkey as the most suitable animal model of human brain organization. The macaque provides the flexibility to explore causal effects by allowing pharmacological, lesion, or even optogenetic interventions that extend well beyond limited case studies and transcranial magnetic stimulation in human subjects (Diester et al., 2011). Additionally, many rodent models and few non-human primate models exist across the spectrum of neurological and psychiatric diseases shown to be accompanied by abnormal functional disruptions. While these models must be interpreted with caution, exploring large-scale topology changes in these models combined with an enhanced understanding of the physiological mechanisms of fluctuation, regulation, and entrainment of LFFs gained from wild-type animals will further RS-fMRI's clinical potential. Following the determination of causal relationships between the disease and altered connectivity patterns, the animal models will also allow the assessment of early diagnostic biomarkers and the development of better drug treatments.

## Future avenues

Resting-state investigations in humans are being conducted at an astonishing pace whereas the same technique is still in its infancy in animal models. Nevertheless, the available reports have presented a promising assessment of preserved attributes upon which multiple research directions can now be developed. It is with the knowledge of normal brain topology and dynamics in the rodent, and even more the macaque that experimental manipulations can be properly interpreted providing new and valuable insights into how the human brain operates across multiple states. In addition to those outlined in the individual sections, below we present additional potential avenues that present an exciting and potentially revealing future for non-human primate (as well as other animals model) investigations.

### Lesions

Functional or structural changes can be induced in the non-human primate with temporary or permanent lesions using techniques such as cooling loops (Lomber et al., 1999), muscimol injections (Dias et al., 1995; Shi et al., 1998), tissue ablation (Rushworth et al., 2003), or optogenetics (Han et al., 2009; Diester et al., 2011). These changes can disrupt local and distributed topology such as small-world topology, particularly when targeting central or provincial hubs. Examining the potentially cascading effects and recovery in a longitudinal model will further provide important insight into the timecourse of network plasticity in response to disruption of brain organization.

### Drugs

Monkeys offer the prospect of conducting controlled drug studies investigating dose-dependent alterations of intrinsic brain connectivity potentially increasing our understanding of their large-scale mechanisms of action. Also, given the extensive documentation on altered connectivity in disease, it is likely that many drugs within therapeutic ranges restore normal connectivity and these changes could be captured in primate disease models.

### Developmental studies

The neural and behavioral development of macaques is well studied and can be directly translated to human timelines (Robinson and Dreher, 1990; Finlay and Darlington, 1995; Clancy et al., 2001, 2007). Long-term developmental studies will not only allow comparison of the network development previously shown in humans (Fransson et al., 2007, 2009), but allow for better temporal resolution of developmental stages from prenatal to adult as well as allow for the incorporation of lesion and drug studies.

### Electrophysiological target identification

The spatial connectivity patterns of RSN nodes can provide future electrophysiological targets that could potentially reveal new functional representations. The multimodal approach would first identify brain areas functionally connected to a region of interest using RS-fMRI. Recording electrodes can then be placed based upon the resulting maps to identify the functional properties of neurons within that area.

### Simultaneous depth recordings

A particularly exciting avenue of future research is the use of multi-site depth recordings combined with simultaneous whole-brain fMRI. Though technically difficult, this will allow for more accurate identification of the neural origins of spontaneous BOLD activity as well as offer insight to the electrophysiological correlates of the dynamic patterns observed in resting-state connectivity (Chang and Glover, 2010; Hutchison et al., 2012b). Beyond their underlying activity and mechanisms, the specific role of low frequency BOLD fluctuations and their synchronization remains an essential avenue of future research that will require extensive work with animal models. Spontaneous activity and dynamic network relationships have emerged as a common theme when studying the brain across multiple spatial and temporal scales in both animals and humans and likely represent a fundamental property of brain organization (Kelso, 1995; Friston, 2000; Rabinovich et al., 2008; Sporns, 2010). The disproportionate metabolic cost driving spontaneous activity further substantiates its role in brain function. Several hypotheses suggesting its role in consolidating, maintaining, and predicting internal and external representations (configurations) have been suggested (Fox and Raichle, 2007) and further empirical evidence is needed. It will also likely be the case that simultaneous depth recordings and fMRI in animal models will be necessary to reveal the origin and potential functional role of anticorrelated areas—a common phenomenon across RS-fMRI investigations. Anticorrelations can occur within or between RSNs and the temporally consistent negative correlations are most often observed between regions lacking direct anatomical connections (Shen et al., in preparation). Whether this often neglected connectivity type represents a form of temporal segregations (Fox et al., 2005) or a manifestation of noise-driven transitions between different meta-states (Deco et al., 2009) could provide further insights into ongoing brain organization.

### Other non-human primate models

While the macaque remains the best-studied non-human primate model, offering a wealth of existing literature upon which to base present resting-state results, this should not exclude the use of other primate models that can circumvent some of its limitations and provide valuable new insights (Tenney et al., 2004). Marmosets in particular offer a model that minimizes some of the cost, handling, and housing requirements of the macaque while still retaining many of its characteristics. These small new world monkeys reproduce faster, expediting developmental studies (Bourne and Rosa, 2006), and have been the model of choice for many systems neuroscientists (Roberts and Wallis, 2000). Perhaps most critically, due to their smaller size, marmosets can be imaged in the smaller bores of higher-field magnets (>9.4 T) allowing for increased spatial resolution that may be necessary for detailed connectivity mapping and exploring the aforementioned variability in neurovascular properties between brain regions.

## Conclusions

Ongoing work using RS-fMRI has demonstrated that brain network topology and its reciprocal temporal features are ubiquitous across mammalian species. Greater brain complexity can also be demonstrated from more distributed network organization and lateralization of functional connectivity patterns. From an applied research perspective, the findings support the use of animal models, particularly non-human primates for the study of network disruptions in disease and exploration of the true underpinnings of the RS-fMRI signal. The applications and future avenues of research extending from current work are broad and will aid in our understanding of normal and abnormal brain function.

### Conflict of interest statement

The authors declare that the research was conducted in the absence of any commercial or financial relationships that could be construed as a potential conflict of interest.
